# Symbiont genotype influences holobiont response to increased temperature

**DOI:** 10.1038/s41598-022-23244-3

**Published:** 2022-11-01

**Authors:** Jennica J. Moffat, Mary Alice Coffroth, Piper D. Wallingford, Casey P. terHorst

**Affiliations:** 1 Department of Biology, California State University, Northridge, CA 91330 USA; 2 Department of Geology, University at Buffalo, Buffalo, NY 14260 USA; 3 The Nature Conservancy, Los Angeles, CA 90071 USA

**Keywords:** Climate-change ecology, Evolutionary ecology, Evolution

## Abstract

As coral reefs face warming oceans and increased coral bleaching, a whitening of the coral due to loss of microalgal endosymbionts, the possibility of evolutionary rescue offers some hope for reef persistence. In tightly linked mutualisms, evolutionary rescue may occur through evolution of the host and/or endosymbionts. Many obligate mutualisms are composed of relatively small, fast-growing symbionts with greater potential to evolve on ecologically relevant time scales than their relatively large, slower growing hosts. Numerous jellyfish species harbor closely related endosymbiont taxa to other cnidarian species such as coral, and are commonly used as a model system for investigating cnidarian mutualisms. We examined the potential for adaptation of the upside-down jellyfish *Cassiopea xamachana* to increased temperature via evolution of its microalgal endosymbiont, *Symbiodinium microadriaticum*. We quantified trait variation among five algal genotypes in response to three temperatures (26 °C, 30 °C, and 32 °C) and fitness of hosts infected with each genotype. All genotypes showed positive growth rates at each temperature, but rates of respiration and photosynthesis decreased with increased temperature. Responses varied among genotypes but were unrelated to genetic similarity. The effect of temperature on asexual reproduction and the timing of development in the host also depended on the genotype of the symbiont. Natural selection could favor different algal genotypes at different temperatures, affecting host fitness. This eco-evolutionary interaction may be a critical component of understanding species resilience in increasingly stressful environments.

## Introduction

To keep pace with rapid climate change and avoid extinction, declining populations must respond rapidly through migration, acclimation, or adaptation via evolutionary changes in traits^1^. Rapid evolutionary responses have been documented in response to strong selection pressures, such as predation^2, 3^ and parasitism^4^, as well as pressures related to climate change, such as increased temperature^5^, more frequent droughts^6, 7^, and shifting seasons^8^. Rapid evolution can be driven by strong selection, but also facilitated by high genetic diversity, large population sizes, and/or short generation times. The evolution of declining populations on time scales likely to affect ecological interactions may rescue populations from extinction if there is sufficient genetic variation on which natural selection can act^9, 10^.

However, species do not exist in isolation, and just as climate change can drive evolutionary change, so can interactions with other species^11^. Climate change can alter evolution in response to species interactions, and conversely, species interactions can alter evolutionary responses to climate change^11–13^. By focusing on individual species in isolation from their community context, we may be overlooking potentially important interactions that could influence how individuals respond to natural selection in a community context^14^. Obligate mutualisms may be particularly vulnerable to environmental change because the fitness of each species is dependent on the performance of its partner. In contrast, adaptation of one partner in an obligate mutualism to environmental change could result in adaptation of both partners. In many obligate mutualisms, one partner is a relatively small, fast-growing microbe with greater evolutionary potential on short time scales than their relatively large, slower growing hosts^15^. In such mutualisms, the rapid evolution of a symbiont could affect the fitness and survival of the host, thus rescuing the holobiont (i.e., host and its associated symbionts). To determine whether such associative evolutionary rescue from climate change is a possibility, we must first investigate whether there is standing genetic variation in (a) the benefits that symbionts can provide to hosts at different temperatures, and (b) how symbionts affect host fitness responses to increased temperature.

Cnidarian-dinoflagellate mutualisms, primarily coral reef hosts and their microalgal endosymbionts in the family Symbiodiniaceae, are frequently studied symbioses because of their essential role in providing habitat in nutrient-poor tropical ecosystems and their fragility in an increasingly stressful oceanic environment^16, 17^. When ocean temperatures exceed a threshold, the mutualism between reef cnidarians and their algal symbionts breaks down, resulting in bleaching. Rising ocean temperatures have resulted in increasing numbers of bleaching events^18–21^, and a one degree Celsius increase of summer maximum temperature can cause mass bleaching^22^. Given the dependence of cnidarian hosts on photosynthetically-derived carbon from their algal endosymbionts, bleaching often results in the death of the host organism^23–25^. Thermal tolerance of the holobiont is at least partly determined by symbiont identity at the genus and species level^26–29^. More recent work has begun to examine how different symbiont genotypes within the same species can affect thermal tolerance of the holobiont^29–35^. Compelling evidence suggests that algal evolution in response to increased temperature may reduce bleaching and confer adaptation of the holobiont to rising ocean temperatures^36–40^.

**Figure 1:**
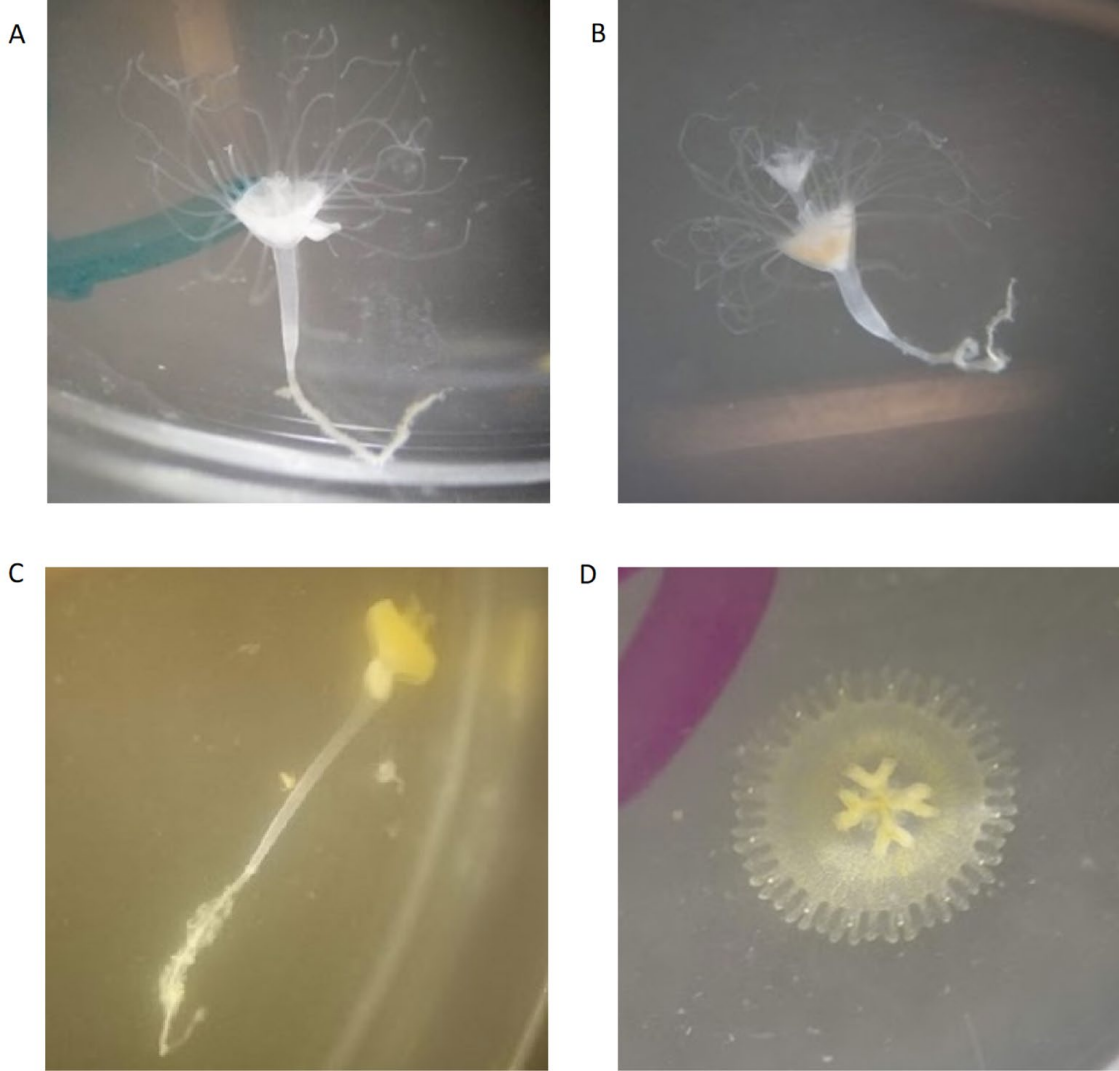
*Cassiopea xamachana* throughout development. (**A**) Budding aposymbiotic polyp, (**B**) infected polyp with brown tint of symbionts, (**C**) polyp in late stages of strobilation, and (**D**) newly detached ephyra.

Due to their long generation times and the difficulty of rearing many coral species in the laboratory, determining the effects of different symbionts on coral fitness can be difficult. Instead, here we use a model cnidarian-dinoflagellate mutualism, the upside-down jellyfish *Cassiopea xamachana* and its algal symbiont, *Symbiodinium microadriaticum*, to investigate the relationship between different algal genotypes and host fitness in response to thermal stress. These hosts are relatively hardy and easy to rear in laboratory conditions and have shorter generation times than most corals. The hosts can be maintained aposymbiotically until they are infected with the appropriate algal strains, making it possible to perform manipulative experiments with symbionts. Like many corals, *Cassiopea* take up algal symbionts when they are in their polyp stage (scyphistoma) and rely on the products of photosynthesis for nutrition^41–43^. However, unlike their coral relatives, *Cassiopea* additionally depend on symbionts as a developmental cue. The scyphistomae do not produce ephyrae, the juvenile stage of their free-swimming jellyfish form, until they obtain algal symbionts^44–46^ (Fig. 1). Once the first ephyra detaches, a polyp can revert to budding or prepare to produce another ephyra^46^. In the absence of algal symbionts, polyps remain in the asexual stage of their life cycle. Ephyrae develop into adult jellyfish and eventually sexually reproduce aposymbiotic planula, which settle on appropriate substrates, such as mangrove leaves. Planulae develop into polyps and reproduce asexually through budding until infected with algal symbionts and begin to strobilate and produce ephyrae.

We used this mutualistic pair to first investigate how (1) the growth rate and physiology of five different algal genotypes respond to temperature in vitro and (2) how these five genotypes affect host fitness components in response to increasing temperature. We quantified several traits likely to affect the interaction between host and symbionts. Photosynthesis and respiration of the algal symbionts are likely to affect symbiont supply of sugars and other nutrients to the host. Symbiont growth rate and carrying capacity (maximum sustainable number of individuals in the population), as measured in vitro, are likely to affect the host, although the direction may be context-dependent. At low symbiont densities, high growth rates or carrying capacities may increase nutrient supply and benefit the host, but symbionts could become more parasitic to hosts at higher densities.

## Results

### Experiment 1: Effects of Temperature on in vitro algal growth and physiology

**Figure 2:**
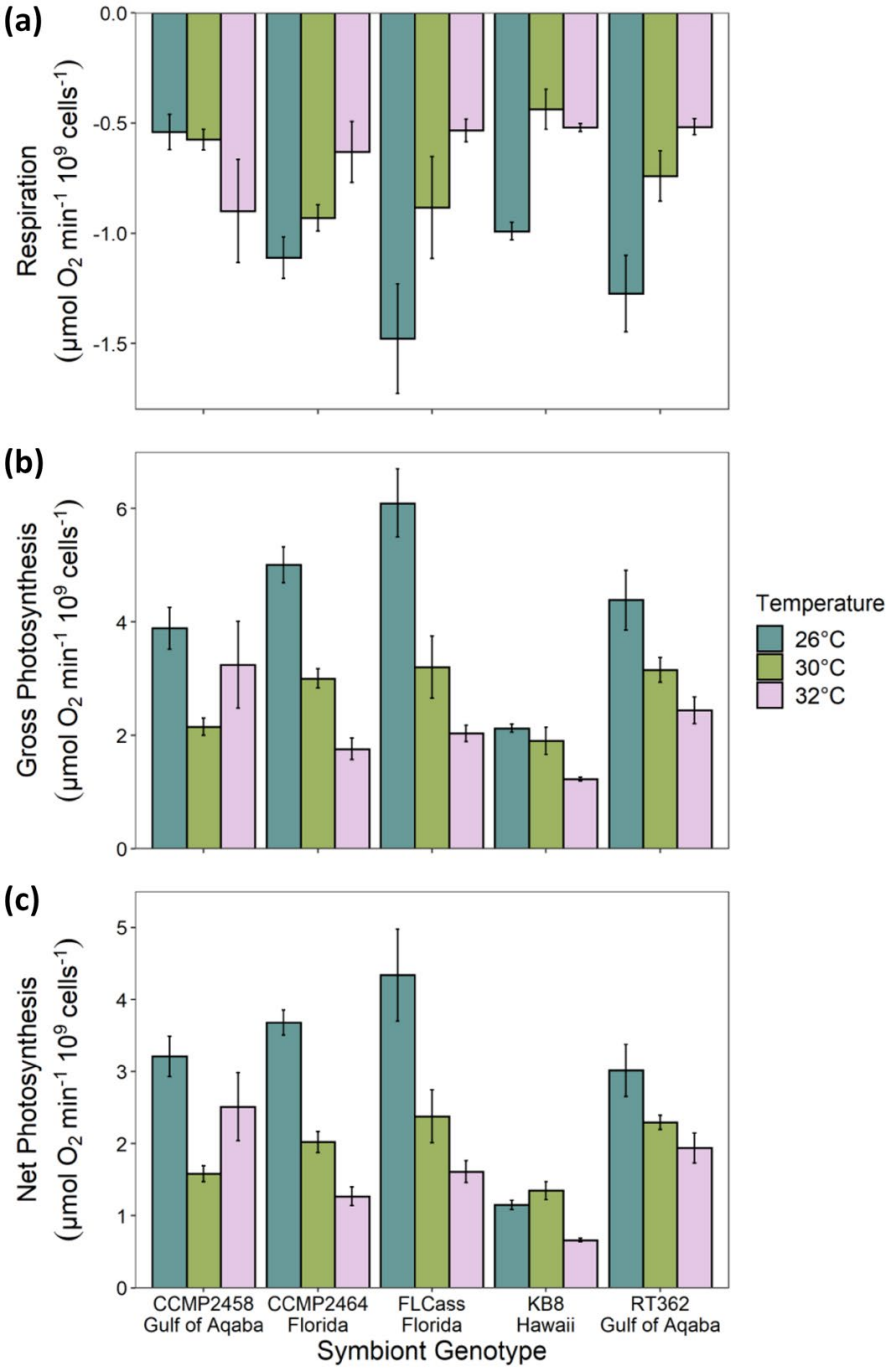
(**a**) Respiration (*P* < 0.001), (**b**) gross photosynthetic (*P* < 0.001), and (**c**) net photosynthetic (*P* < 0.001) rates of five symbiont genotypes measured in vitro at 26 °C, 30 °C, and 32 °C. Values represent mean ± SE, n = 4.

We found a significant interaction between temperature and genotype on respiration (F_8,45_ = 4.56, P < 0.001), gross photosynthesis (F_8,45_ = 4.71, P < 0.001), and net photosynthesis (F_8,45_ = 6.51, P < 0.001) of algae grown in culture. Most cultures had lower respiration at higher temperatures, although the magnitude of the response varied among genotypes (Fig. 2), with FLCass demonstrating the largest decrease in average respiration from 26 °C to 32 °C of 64% compared to CCMP2464’s decrease of 43%. However, one genotype (CCMP2458) showed its highest respiration rate at 32 °C. Similarly, both net and gross photosynthesis generally decreased at higher temperatures, but the magnitude of the response varied among genotypes; genotype CCMP2458, however, showed a non-linear response of photosynthesis to increasing temperature (Fig. 2).

**Figure 3:**
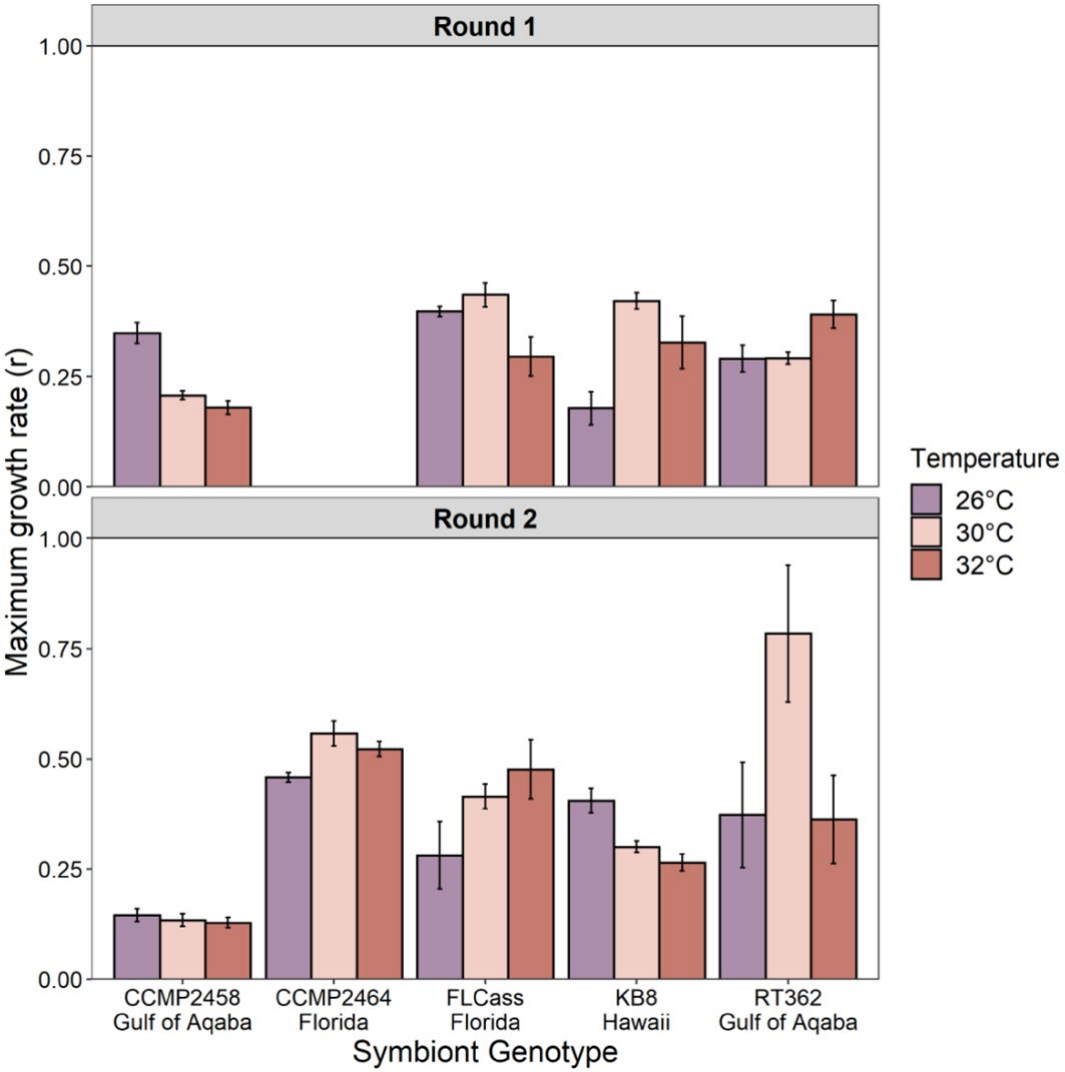
Mean maximum growth rate (+ SE) of symbiont genotypes measured in vitro at three experimental temperatures during each experimental round. CCMP2464 did not grow during Round 1.

We found significant differences in growth rate parameters between rounds of the experiment. However, in both cases, we found a significant interaction between temperature and genotype on maximum growth rate in culture (Fig. 3; Round 1: F_6,36_ = 10.8, P < 0.001; Round 2: F_6,36_ = 2.23, P = 0.04). In both rounds, genotypes showed variation in their response to temperature, with some genotypes increasing their growth rate in response to an increase in temperature from 26 °C (two genotypes in Round 1; three genotypes in Round 2), and other genotypes showing decreases in growth rate with increasing temperature (two genotypes each in Rounds 1 and 2) (Fig. 3).

We found variation in relatedness among our symbiont genotypes (Table S1, F_ST_ range = 0.144–0.238), which may reflect differences in collection area, as well as time spent in culture (up to 15 years in some cases). Genetic relatedness was significantly positively correlated with differences in carrying capacity (K) among algal genotypes in round 2 of the growth experiment at 26 °C (r = 0.624, P = 0.033) and 32 °C (r = 0.709, P = 0.017), but not at 30 °C (r = 0.127, P = 0.040). In the first round of the experiment, there was not a significant relationship between differences in K and F_ST_ at any of the three temperatures (Table S2). Mantel tests revealed no other significant correlations between F_ST_ and differences in respiration, gross or net photosynthesis, or growth rate at 26 °C (P > 0.17), 30 °C (P > 0.23), or 32 °C (P > 0.20) (Table S2).

### Experiment 2: Effects of symbionts on host fitness and physiology in response to temperature

**Figure 4:**
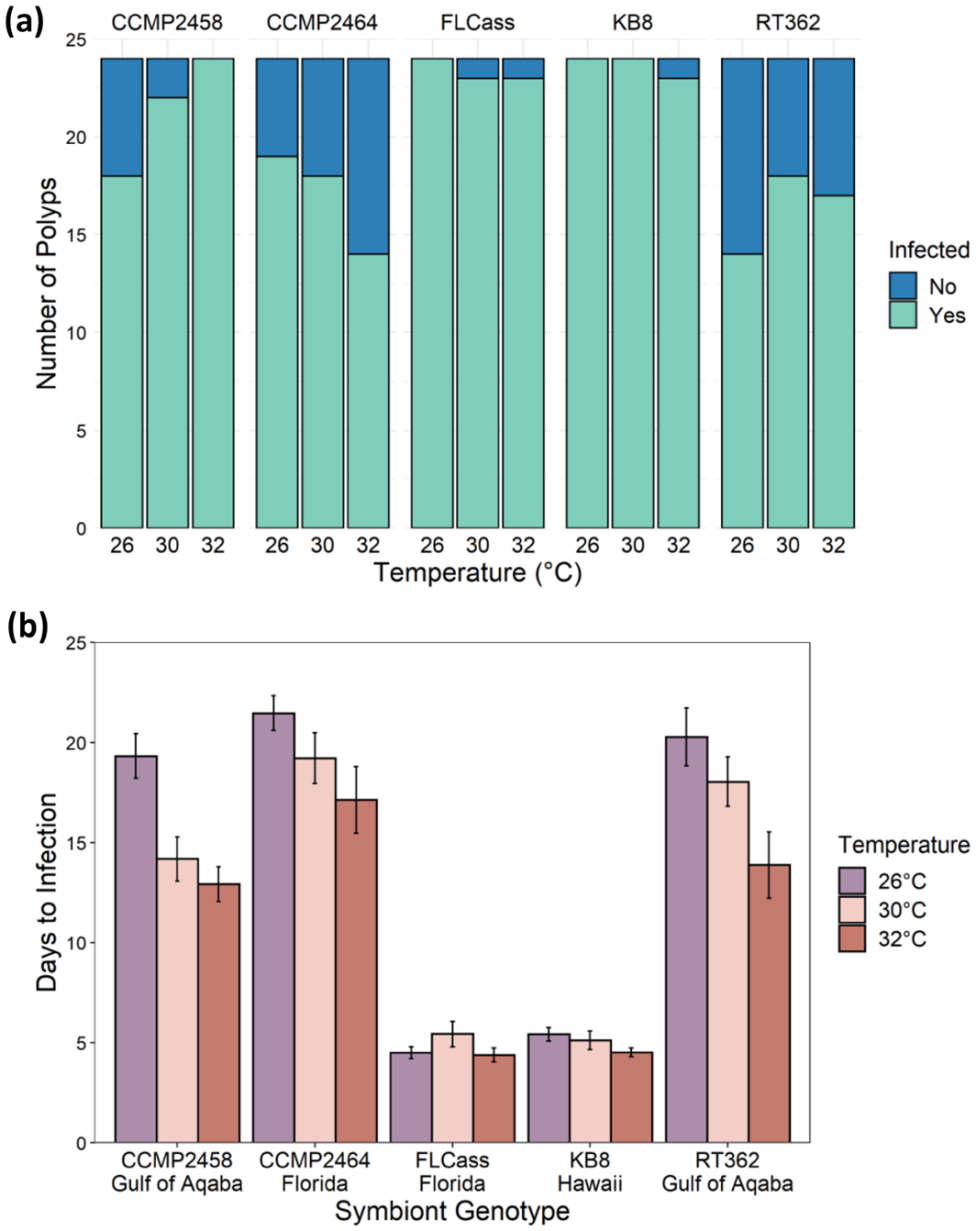
(**a**) Number of polyps that were infected with one of five algal genotypes at each temperature by the end of the experiment. (**b**) Mean (+ SE) days that it took for polyps to become infected with one of five algal genotypes across the three experimental temperatures.

**Figure 5:**
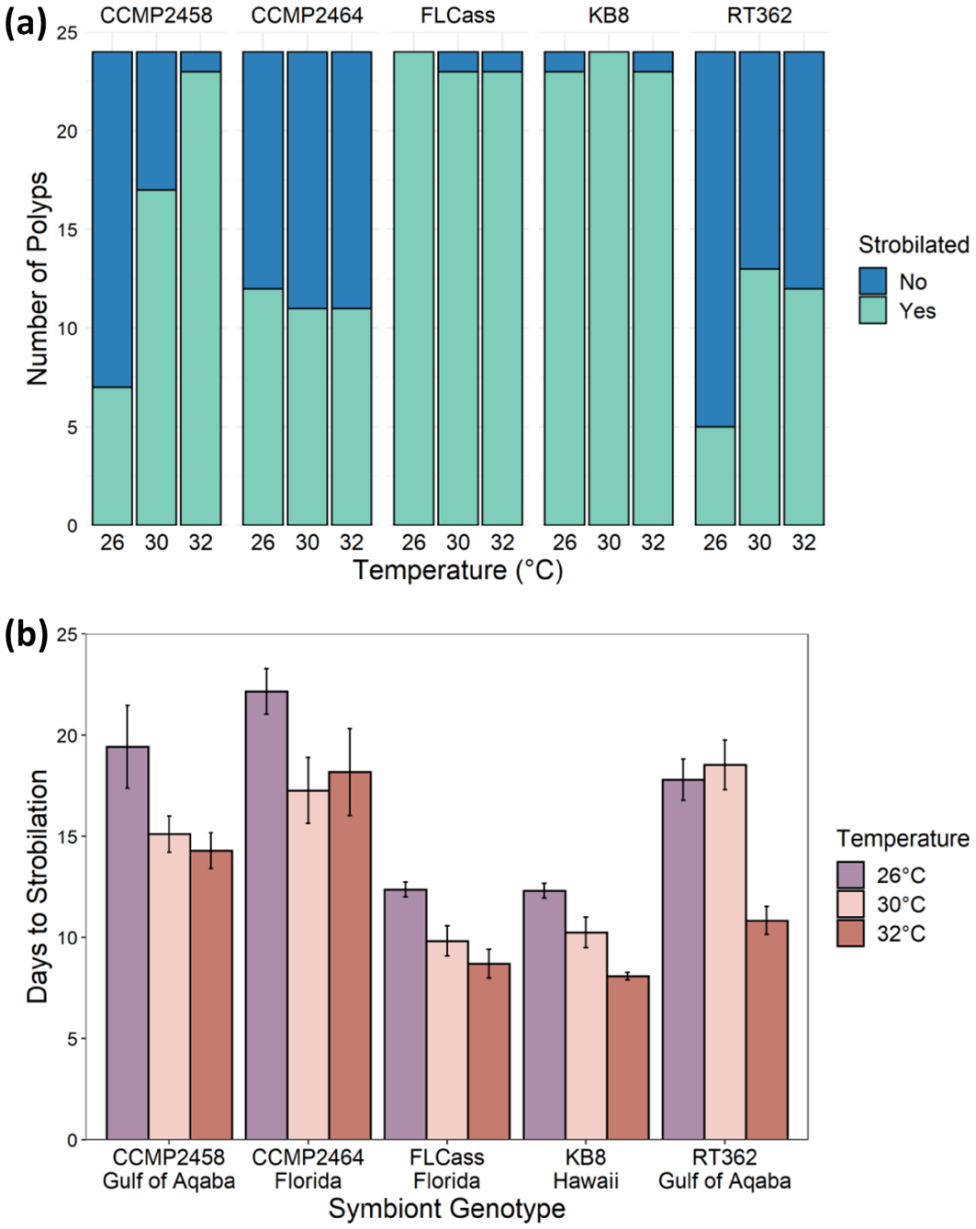
(**a**) Number of polyps infected with one of five algal genotypes that began to strobilate at each temperature by the end of the experiment. (**b**) Mean (+ SE) days that it took for polyps infected with one of five algal genotypes to begin to strobilate at each temperature.

**Figure 6:**
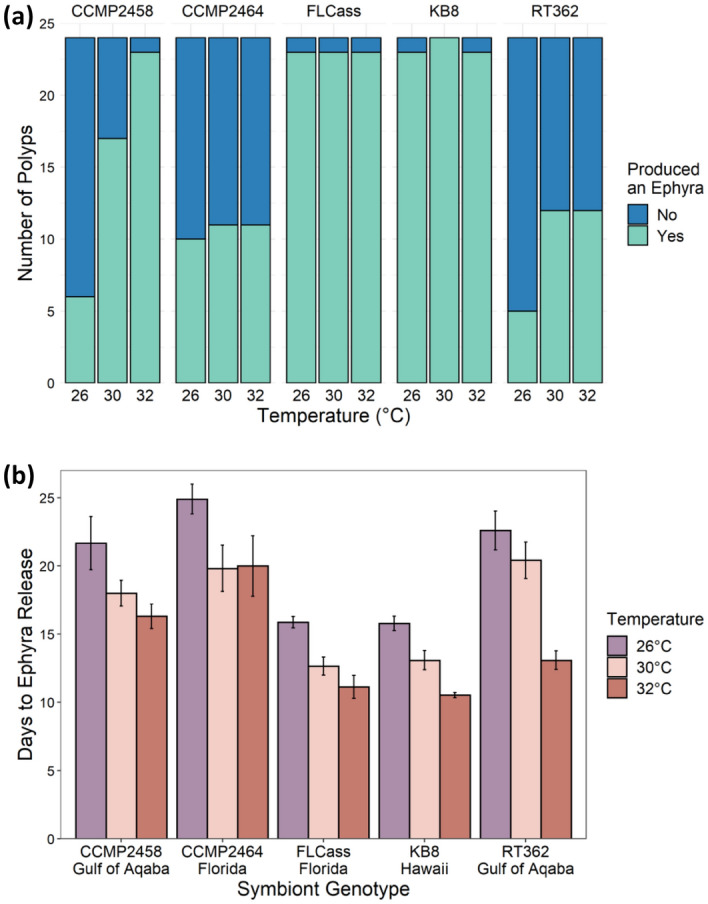
(**a**) Number of strobilating polyps that produced an ephyra by the end of the experiment when infected with one of five algal genotypes at each temperature. (**b**) Mean (+ SE) days that it took for infected strobilating polyps to release their first ephyra when hosting one of five algal genotypes at each temperature.

Nearly all polyps survived until the end of the experiment. Of the 23 polyps that died, 13 died after producing an ephyra and 10 died before producing an ephyra, so 98% of the polyps survived until the end of the experiment or only died after reproduction, which we counted as survival. Survival was not dependent on temperature (χ^2^ = 1.65, df = 2, P = 0.438), genotype (χ^2^ = 3.30, df = 5, P = 0.654), or their interaction (χ^2^ = 7.86, df = 10, P = 0.642). However, there was a significant interaction between the effects of algal genotype and temperature on whether polyps reached particular stages of development: infected (χ^2^ = 16.4, df = 8, P = 0.038; Fig. 4a), strobilation (χ^2^ = 23.5, df = 8, P = 0.003, Fig. 5a), and ephyra production (χ^2^ = 18.7, df = 8, P = 0.016; Fig. 6a). Additionally, for individuals that reached each stage, there was a significant interaction between the effects of algal genotype and temperature on the number of days until successful infection (F_8,290_ = 2.48, P = 0.013; Fig. 4b) and the beginning of strobilation (F_8,236_ = 2.18, P = 0.029; Fig. 5b). For the time to ephyra release, there was not a significant effect of the temperature by genotype interaction (F_8,231_ = 1.65, P = 0.111), but there were independent effects of both temperature (F_2,231_ = 4.76, P = 0.009) and genotype (F_4,231_ = 12.3, P < 0.001) (Fig. 6b). For two symbiont genotypes (FLCass and KB8), polyps become infected (approximately 3–4.7 times faster than other genotypes across all temperatures) and began strobilating (approximately 1.2–2.3 times faster than other genotypes at 32 °C, 1.5–1.9 times faster at 30 °C, and 1.4–1.8 at 26 °C) much earlier than when hosting one of the other three symbiont genotypes, and therefore nearly all of the polyps hosting these two genotypes of symbionts reached all three stages of development faster (Figs. 4b, 5b, 6b). The polyps hosting the other three symbiont genotypes generally developed faster at 30 °C or 32 °C than 26 °C, although to varying degrees.

Temperature and algal genotype affected the response of other proxies for host fitness. We found a significant genotype by temperature effect on bud production (F_10,413_ = 2.80, P = 0.002) with some algal genotypes (CCMP2464, KB8, RT362) resulting in decreased bud production with temperature, similar to the aposymbiotic control treatment (Fig. 8a). However, the magnitude of decrease in response to temperature varied among those genotypes (Aposymbiotic polyps showed the smallest difference between 32° and 26 °C, with a 26% decrease in bud production, compared to RT362 which had the greatest change at 61% decrease in bud production from 32° to 26 °C). Other algal genotypes resulted in non-linear (CCMP2458) or little response (FLCass) of bud production to increasing temperature (Fig. 8a). The uninfected controls produced more buds that infected polyps but did not strobilate or produce any ephyrae (Fig. 8a). We found a significant interaction between algal genotype and temperature on ephyra production per polyp (F_8,344_ = 3.38, P < 0.001). Three algal genotypes (CCMP2458, FLCass, and RT362) resulted in increased ephyra production with increasing temperature, although to various degrees, with the magnitude of change for these three genotypes ranging from 1.4 to 3.8 times as many ephyra from 26 °C to 32 °C (Fig. 8b). One genotype (CCMP2464) showed little response of ephyra production to increasing temperature and one genotype (KB8) showed a non-linear pattern with temperature (Fig. 8b).

We found no effect of genetic relatedness among symbiont genotypes (Fst) on differences in rates of infection, strobilation, ephyra production, or the number of buds or ephyra produced at 26 °C (P > 0.15), 30 °C (P > 0.21), or 32 °C (P > 0.29) (Table S2).

## Discussion

There has been considerable work demonstrating that different genera or species of algal symbionts affect performance of their cnidarian hosts and the response of the holobiont to increased temperature^37, 47–54^. As in previous research^29, 30^, we found similar effects of genetic diversity at a lower taxonomic level—different genotypes within the species *Symbiodinium microadriaticum* have different physiological responses to temperature and differentially affect host fitness responses to temperature. The algal-cnidarian mutualism is largely based on the amount of photosynthetically-derived sugar that the algae provide to their host, often providing up to 95% of the nutrition to the host in nutrient-poor water^55, 56^. Here we found that the algal genotype with the greatest potential to provide benefits to the host depends on temperature, as the response of respiration and photosynthesis to temperature differs among algal genotypes. Algal genotypes also have different effects on the developmental timing and fitness of *Cassiopea xamachana* hosts. Understanding the ecological dynamics of this holobiont in response to increasing ocean temperatures will require understanding selection on and evolutionary dynamics of the algal symbionts.

### Variation in algal physiology in response to temperature

We measured algal traits that are likely to affect thermal tolerance of the holobiont^33^: photosynthesis, respiration, and growth rates. Previous work has identified tremendous variation in thermotolerance of these traits among and within species in the family Symbiodiniaceae^32–34, 57–59^. Despite variation in growth rates at different temperatures, the fact that all genotypes had positive growth rates at temperatures ranging from 26 °C to 32 °C, suggests broad thermal tolerance in *Symbiodinium microadriaticum*. However, despite this high level of tolerance relative to other species, four of the five *S. microadriaticum* genotypes in our study showed decreased rates of respiration and photosynthesis with increased temperature, indicating variation in the amount of nutrients that could be supplied to hosts. The genotypes with the highest growth and photosynthetic rates at 26 °C did not have the highest growth and photosynthetic rates at 30 °C or 32 °C (Figs. 2, 3). These trends suggest that, as ocean temperatures rise, the particular genotypes with the highest relative fitness that experience positive selection will differ among temperatures, resulting in selection for more heat tolerant genotypes at higher temperatures. There is evidence for rapid evolution in other Symbiodiniaceae species^38, 39^, so evolution of symbionts on ecological time scales may occur commonly in cnidarian-algal symbioses and provide the potential for evolutionary rescue of these important mutualisms^60, 61^.

Whether differences in thermal tolerance among algal genotypes allow for temperature to impose selection on thermal tolerance depends on whether such variation exists within an algal population. Our results serve as a proof of concept that such dynamics are possible in cnidarian-algal mutualisms. However, the genetic diversity in our experiment arises from *S. microadriaticum* genotypes collected from the Pacific, Atlantic, and Indian Oceans, and from multiple host species (Table 1), although these host populations may all belong to one lineage^62^. Whether such variation also exists within a population remains to be seen. The lack of any relationship between genetic similarity and trait similarity across our global sample though, suggests that even in closely related populations of algae, there may still be sufficient trait variation upon which selection can act. Whether such variation need exist within a single polyp, a population of polyps, or in the free-living stage of the algal life cycle, will depend on how often hosts exchange symbionts with the water column and how far algae travel in currents during the free-living phase of their life cycle.

The effect of temperature on symbiont growth rate not only varied between genotypes, but also between experimental rounds (Fig. 3, Fig. S1). Both rounds of the experiment were conducted in seemingly identical controlled conditions in growth chambers. However, the length of the experiment differed between rounds. The six fewer days of growth in the second round resulted in many cultures still in their exponential phase of growth, before they had reached a carrying capacity (Fig. S1). Logistic growth curves fit less well in the second round, relative to the first, suggesting that the estimates of population growth parameters in the first round are more precise. Despite the variation between rounds, overall, the variation in growth rates between genotypes suggests that populations of *S. microadriaticum* exhibit varying responses to temperature stress, suggesting sufficient opportunity for selection. The amount of time that cultures spend in an exponential growth phase versus the amount of time they spend close to their carrying capacity could affect selection on growth rate in vitro. Cultures maintained in exponential growth conditions may experience selection for increased growth rate, but those maintained in steady state growth may experience selection for higher carrying capacities. Past work suggests little, if any, correlation between these traits in symbiont species^35^, but the fact that we observed variation in genotype responses to temperature adds generality to our results. If the goal of artificial selection is to select for or against growth rates, then the population dynamics in vitro and the timing at which cultures are refreshed may be important.

### Host response to temperature is dependent on symbiont genotype

Algal genotype had a significant effect on the response to temperature of a number of the fitness components of the host. *Cassiopea xamachana* can respond to changes in temperature through changes in growth rate and respiration rate^63, 64^, budding rate^65^, and developmental timing^62, 63^. Our results confirm that infection, budding, and developmental timing are affected by temperature, but importantly, the responses to temperature are also affected by the genotype of the algal symbionts. As the genetic composition of the symbiont community changes, the capacity for the holobiont to respond to changes in temperature is likely to change as well.

Developmental rate is an important component of fitness, with faster development times potentially resulting in higher reproductive output via sexual reproduction^66, 67^. However, investment in sexual reproduction potentially trades-off with investment in asexual reproduction via budding. Budding asexually produces more polyps, but strobilation and ephyra production eventually allow for sexual reproduction in mature jellyfish. Because *Cassiopea*, once infected, shifts energy from asexual budding to strobilation^68^, the polyps that developed fastest produced the most ephyrae, but the fewest buds, resulting in a significant, albeit weak, correlation between ephyrae and buds (Kendall’s Tau = − 0.14, P = 0.003). This suggests multiple pathways to increased fitness, but only one of them involves algal symbionts. Although algal symbionts are required for the host to complete its life cycle, a longer time spent without algal symbionts is likely to result in greater asexual reproduction via budding (Fig. 8a) and may eventually pay off with higher overall capacity for sexual reproduction by more polyps in the future, although polyps in nature are often quickly infected with a diversity of symbionts^69, 70^.

**Figure 7:**
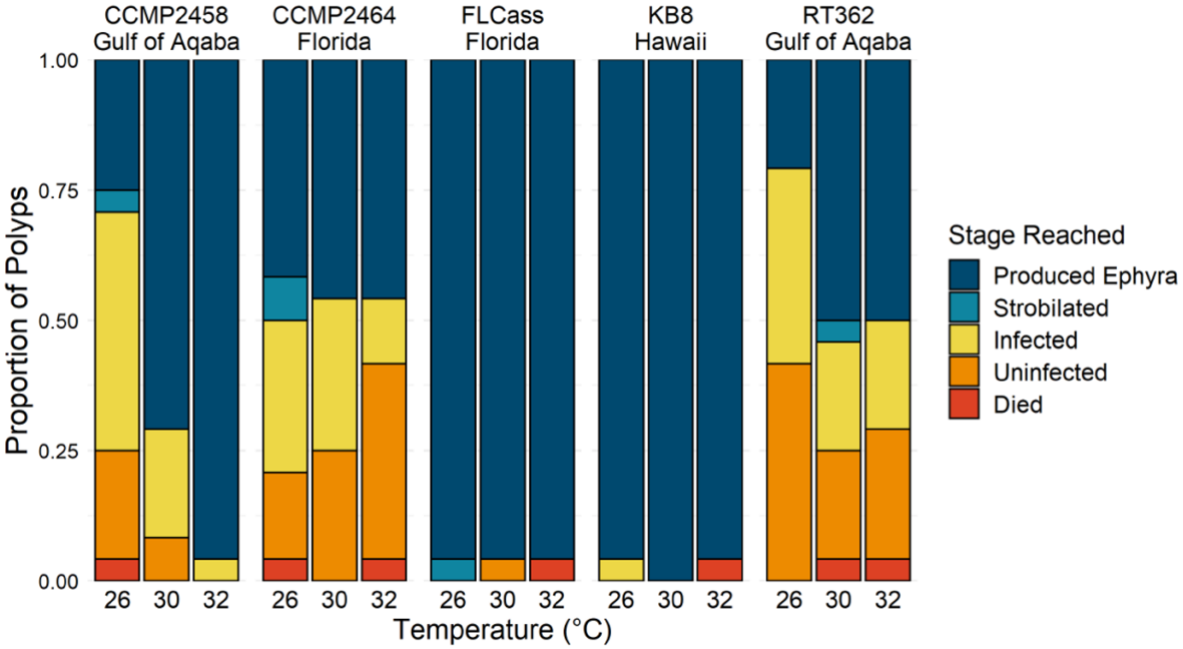
Proportion of polyps in each stage of development that died before infection, were uninfected or infected, strobilated, or released an ephyra at the end of the experiment.

**Figure 8:**
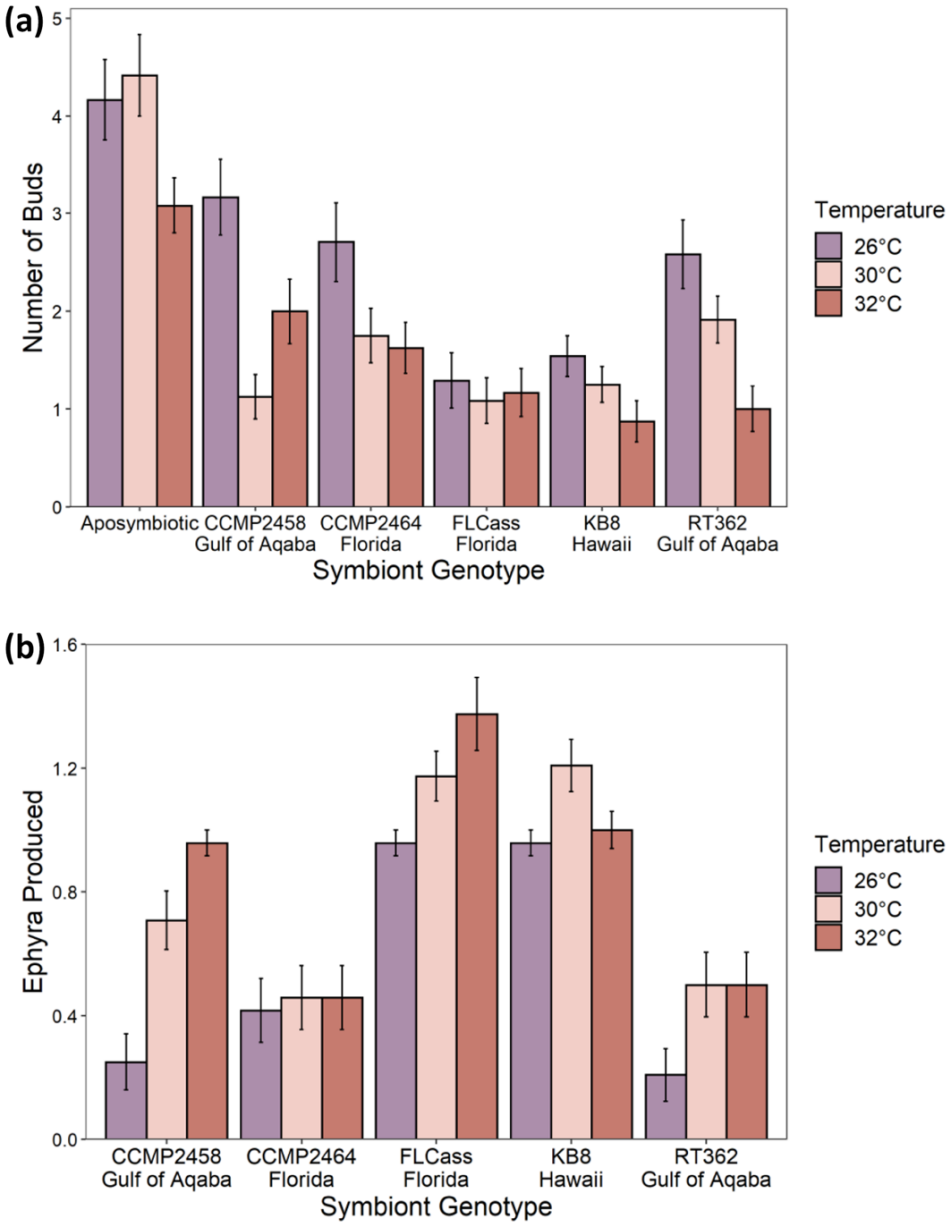
(**a**) Mean (+ SE) total bud production for aposymbiotic polyps and polyps infected with one of five algal genotypes across the three experimental temperatures. (**b**) Mean (+ SE) ephyra production per polyp infected with one of five algal genotypes at each temperature.

Across the three temperatures, polyps hosting either FLCass or KB8 generally developed faster than polyps hosting any of the other three symbiont genotypes, which resulted in a higher proportion of polyps reaching all three stages of development and ultimately greater ephyra production (Fig. 7). For polyps hosting these two symbiont genotypes, increased temperature decreased the time to strobilation and ephyra release, with more rapid ephyra production at higher temperatures. Subsequently, polyps hosting either FLCass or KB8 at the two higher temperatures were the only groups that produced more than one ephyra. In contrast, polyps hosting CCMP2458 and RT362 produced the fewest ephyra at 26 °C, but also produced many buds at that temperature, likely due to fewer polyps being infected at 26 °C. As temperature increased, polyps hosting those two genotypes showed increased infection rates and ephyra production, but decreased bud production. Polyps hosting CCMP2464 also had higher rates of infection and lower bud production with increased temperature, however this response did not result in an increase of ephyra production, which remained consistently low, relative to polyps hosting other symbiont genotypes, across temperatures (Fig. 8). Ultimately, determining the effects of each algal genotype on lifetime fitness of the host will depend on the survival rate of polyps produced via budding, ephyra production by those polyps, and successful sexual reproduction by adult jellyfish. However, the variable temperature responses of each host-symbiont combination suggests that genetic variation of the symbiont is likely to play an important role in lifetime reproductive success.

Interestingly, the magnitude of algal trait responses to temperature in vitro was not a good predictor of the magnitude of the response of hosts to temperature. For example, net photosynthesis of FLCass decreased sharply in response to increased temperature in vitro (Fig. 2), but temperature had little effect on the time to infection or number of buds produced by hosts (Figs. 4, 8). Polyps infected with FLCass actually produced more ephyra with increasing temperature (Fig. 6), despite the decrease in potential benefits provided by the symbionts, as measured in vitro (Fig. 2). We found little evidence that traits measured in vitro (respiration, photosynthesis, growth rate) were correlated with any aspect of host fitness at 26 °C (P > 0.18), 30 °C (P > 0.27), or 32 °C (P > 0.14), with two exceptions: at 30 °C, in vitro growth rate was positively correlated with bud production (Mantel r = 0.64, P = 0.033) and at 32 °C, respiration rate was positively correlated with bud production (Mantel r = 0.84, P = 0.017). Given the large number of possible potential correlations, we are cautious about giving too much weight to these lone two significant correlations, but future research could investigate why these correlations might only exist at these temperatures. Overall, these results demonstrate the difficulty of predicting holobiont fitness and temperature response directly from in vitro traits of symbionts^35, 38^ and suggest that individual interactions between hosts and symbionts can produce unique holobiont responses, which may be of more importance for understanding holobiont performance^29, 30^.

The effect of algal genotype on host fitness responses to increasing temperature may be due more to compatibility between host and symbiont genotypes, rather than specific physiological differences among symbiont genotypes. Genotypes CCMP2464 and FLCass were collected from *C. xamachana* individuals in Florida, but the other genotypes were collected from other hosts. Although all symbiont genotypes belong to the same species, these two genotypes may be locally adapted to their hosts that also originated in Florida. However, infection times varied among genotypes more broadly (Fig. 4), so host-symbiont compatibility also does not seem to explain the host response to temperature entirely either.

The lack of correlation between symbiont traits measured in vitro and host fitness responses to temperature does not mean that these trait measurements are irrelevant though. Species in the family Symbiodiniaceae spend a portion of their life cycle in the ambient environment^71^, where genetic and trait variation and selection may differ from selection *in hospite*. The evolution of symbiont populations in the ambient environment could influence host fitness as *Cassiopea* and many cnidarian species take up environmental symbiont populations every generation, or show changes in algal communities following bleaching events^49, 72^. Additionally, because species in the family Symbiodiniaceae have a relatively high mutation rate^60^, they could continue to accumulate variation, even *in hospite*. The effects of different selection pressures on populations of symbionts in vitro versus *in hospite* is important for understanding the eco-evolutionary dynamics of these mutualisms.

### Implications for evolutionary rescue via mutualists

As corals experience massive bleaching events and die-offs^17^, there has been a large focus on the potential for the holobiont to be rescued by hosting more thermally tolerant symbionts^26, 37, 39, 48, 60, 61, 73^. Although thermally tolerant symbionts can be acquired via exchange with the ambient environment^72, 74, 75^, cnidarian-algal mutualisms are often very specific and switching to alternate taxa may not be feasible^76, 77^. Early polyp stages of *Cassiopea* can be flexible in symbiont uptake and host several species, but strobilation only occurs with a smaller subset of taxa^45, 54, 70^. *Cassiopea xamachana* adults almost exclusively host *Symbiodinium microadriaticum* and polyps will preferentially take up homologous strains when offered homologous and heterologous strains simultaneously^70^.

Our results suggest the potential for evolution of symbiont populations that could occur within or outside the host. However, because in vitro traits do not explain holobiont fitness well, from a conservation standpoint, it may not be productive to conduct selection experiments on symbionts in vitro, but rather to focus on selection on holobionts and consider evolution in this community context^14^. Algal symbionts have a high mutation rate, so selection could act on favorable mutations and result in evolution of thermal tolerance *in hospite*^60^. If algal thermal tolerance evolves in the ambient environment, or in populations of algae *in hospite* that are later expelled, then symbiont switching could play a role in acquiring temperature tolerant genotypes while maintaining the specificity of the mutualism. If the evolution of thermal tolerance in algal populations confers increased holobiont thermal tolerance, then perhaps the holobiont could experience evolutionary rescue via association with symbionts. Fully answering this question will require tracking lifetime fitness of the host and quantifying benefits and costs of hosting thermally tolerant symbionts. Hosting thermally-tolerant symbionts can come with costs at less stressful temperatures^78–80^, so understanding lifetime fitness is critical. The genetic composition and evolution of algal symbiont populations seem likely to play a significant role in the response of cnidarians to rising ocean temperatures associated with climate change.

## Methods

We obtained five genotypes of *Symbiodinium microadriaticum* isolated from *Cassiopea* species from Dr. Todd LaJeunesse’s laboratory at Pennsylvania State University and the BURR culture collection in Dr. Mary Alice Coffroth’s lab at University at Buffalo (Table 1). We maintained cultures of each genotype in autoclaved flasks containing approximately 75 mL of f/2 media (Guillard and Ryther 1962) and plugged with a foam stopper. Stock cultures were maintained at 26 °C in a growth chamber with a 14:10 light:dark cycle for several months prior to the start of experiments. We restarted stock cultures every month by inoculating fresh media with ~ 1 mL of the old stock.

DNA was extracted from stock algal cultures using a ZymoBiomics (Irvine, CA, USA) DNA Miniprep Kit, according to manufacturer protocols. ddRad sequencing and processing through a bioinformatics pipeline was performed by Admera Health (South Plainfield, NJ, USA), facilitated through GenoHub (genohub.com). Sequencing produced 3.96 × 10^6^ sequences with an average length of 143 bp that were used to assign haplotype pairwise F_ST_ values of genetic differences among algal genotypes.

We obtained aposymbiotic polyps of *Cassiopea xamachana* from Dr. Mónica Medina’s lab at Pennsylvania State University. All polyps were asexually-produced clones. Over the first two weeks, we transferred polyps twice to progressively larger containers to eventually acclimate them to a 10 L aquarium tank filled with 36 ppt Instant Ocean artificial seawater (Blacksburg, Virginia, USA) at 26 °C with one bubbler. To minimize unintentional algae growth, we maintained the tank in dark conditions, lined with black aquarium-safe plastic lining, and covered with a box. We fed polyps *Artemia* nauplii five days a week and cleaned the tank by performing half-tank water changes with clean artificial seawater weekly.

### Experiment 1: Effects of temperature on algal growth and physiology in culture

To investigate the growth and physiological response of isolated symbiont genotypes to temperature, in July 2019, we grew replicate cultures of each of the five genotypes in growth chambers set to 26 °C, 30 °C, and 32 °C. The mean temperatures (+/− 1 s.d.) in the three chambers were 25.5 °C (+/− 0.5), 30.1 °C (+/− 0.3), and 31.6 °C (+/− 0.2). We initiated 12 replicate cultures of each genotype in sterile flasks with 75 mL of sterile f/2 media with 750,000 total cells (initial density = 10,000 cells/mL) from the appropriate stock culture. Replicate cultures of each genotype were randomly distributed among three identical growth chambers (Percival I-36LLVL) at each of the three temperatures (n = 4 replicate cultures of each genotype at each temperature). We systematically rotated the position of cultures in the growth chamber daily to minimize the effect of any small differences in light and temperature within the chamber. Lights were set on a 12:12 day:night cycle, with an average illumination during the day of 4533 (+/− 456) Lux (approximately 63 µmole m^-2^ s^-1^ based on a conversion of 1 lx = 0.014 µmole m^−2^ s^−1^). We quantified the density of cells in each culture three times per week using the average of four replicate hemacytometer counts. We fit a growth curve to the density of cells in each replicate over 20 days using the *growthcurver* package^81^ in R (v. 4.0.3) and extracted the maximum growth rate (r).

In October 2019, we repeated the same experiment with the same stocks maintained at 26 °C. After three weeks of growth at the three treatment temperatures, we measured photosynthesis and respiration of replicate cultures of each genotype at each temperature using a SDR SensorDish Reader (Loligo Systems, Viborg, Denmark). We filled two wells with 2 mL sampled from each culture and filled two wells with DI water as controls. We placed plates in each of the three growth chambers set to 26 °C, 30 °C, and 32 °C and dark-acclimated plates for five minutes before measuring oxygen concentration every 15 s for 15 min in the dark. We then turned on the lights in each growth chamber and measured oxygen concentration again in the same way. We also quantified algal density using the average of four replicate hemocytometer counts.

We calculated respiration rates for each well as the slope of the best-fit linear line to the decline in oxygen concentration over time in the dark. We subtracted the slope of the same fit in the control wells to account for any background noise. We averaged the two replicate wells for each culture and standardized respiration by cell number. We determined net photosynthesis in the same manner, using the slope of the best-fit linear line to the increase in oxygen concentration over time. Finally, we calculated gross photosynthesis by adding the absolute value of respiration to net photosynthesis for each culture.

### Experiment 2: Effects of symbionts on host fitness and physiology in response to temperature

To investigate how symbiont genotype affects host fitness components in response to increasing temperature, we inoculated aposymbiotic *Cassiopea xamachana* clones from a single isoclonal line with one of the five genotypes of *S. microadriaticum* and maintained them at 26 °C, 30 °C, and 32 °C for 28 days. In addition, we maintained replicates of aposymbiotic polyps at each temperature as a control. In December 2019, we transferred individual polyps of similar size from the stock aquarium into a well of a 6-well cell culture plate with approximately 6 mL of fresh artificial sea water and 6 mL of the stock aquarium water. All polyps were maintained as aposymbiotic in growth chambers for four days, and fed five to eight *Artemia* nauplii once, to allow them to acclimate to their respective temperatures. Throughout the experiment, we fed each polyp five to eight *Artemia* nauplii every third day and performed a water change the following day by removing half of the water and replacing it with fresh artificial salt water. Throughout the experiment, the position of all plates was rotated daily to minimize any minor differences in light and temperature within the growth chamber. We checked the salinity of each well every day and added DI water when needed to maintain the salinity at 36 ppt.

After four days of acclimation to temperature (i.e., Day 1), we supplied polyps with access to one of the five genotypes of *S. microadriaticum*, while also maintaining control polyps with no symbionts. Each well was inoculated with 24,000 cells (2,000 cells/mL) from the appropriate stock algal culture^70^. We fed polyps with *Artemia* nauplii immediately prior to each symbiont inoculation because symbiont uptake occurs more readily when polyps are feeding^70^. We inoculated four six-well plates with each of the five algal genotypes, plus a no algae control, at each temperature, resulting in 24 replicate wells for each genotype by temperature combination (N = 432 polyps). We inoculated wells on days 1, 4, 11, 17, 20, and 23 using the same density of cells from the same stock cultures each time.

For 26 days, we measured the survival of each polyp, as well as asexual reproduction and developmental timing. We visually inspected polyps daily under a dissecting microscope to determine survival. When a polyp died, we emptied the well. We quantified two strategies of asexual reproduction: the total number of buds produced and total number of ephyrae produced. Buds that were produced during the experiment remained in the well; most buds settled and metamorphosed into polyps, but the experiment was not long enough to allow any newly produced buds to become infected and strobilate. We removed all ephyrae that were produced during the experiment the day they detached from the parent polyp. We also measured three developmental timing events: time to visible infection, time to strobilation, and time to ephyra release. Aposymbiotic polyps were white (Fig. 1A) and appeared brown once infected with algae, so polyps were considered infected when a brown tint was observable under the dissecting microscope (Fig. 1B). Polyps were considered to have begun strobilating when they became disc-shaped rather than cone-shaped (Fig. 1C). The time to ephyra release was marked as the day that the ephyra detached from the parent bud (Fig. 1D).

### Statistical analysis

For experiment 1, we used multiple general linear models with Type III SS to test for the effects of algal genotype, temperature, and their interaction on respiration, gross photosynthesis, net photosynthesis, and maximum growth rate. All variables were transformed to meet assumptions of normality and homoscedasticity (respiration: cube root; gross and net photosynthesis: fourth root; growth rate: log). We also used Mantel tests to determine whether genetic relatedness (pairwise F_st_ values) explained differences between genotypes in the means of each of the four traits we measured at each of the three temperatures, using Spearman rank correlations.

For experiment 2, we used multiple general linear mixed effects models to test for the effects of algal genotype, temperature, and their interaction on total bud production and total ephyra production. Bud production data met assumptions of normality and homoscedasticity and ephyra production was square-root transformed to meet assumptions. Because *Cassiopea* will not produce ephyra without symbionts, we removed the aposymbiotic group for analyses of ephyra production and the developmental timing events below.

Because not all polyps reached each developmental stage, we used a hurdle model approach to examine whether algal genotypes, temperature, and their interaction affected (a) development to each subsequent development stage (survival, successful infection, strobilation, and ephyra production) and (b) the time for successful individuals to reach that stage. To determine the effects of each factor on reaching each developmental stage, we used generalized linear models with a binomial error distribution, removing individuals who did not reach a previous stage when analyzing progress to the next stage. Then we used additional general linear mixed effects models to test the effects of the same factors on the time to reach each stage, again removing individuals that did not reach a particular stage. Data were transformed to meet model assumptions (infected and strobilation: log; ephyra: Box-Cox transformation). To account for any variation among replicate plates, in all models we included plate as a random effect, but removed the random effect when it did not increase model fit (determined by AIC). All models were fit using lm or lmer (or glm and glmer for binomial error distributions) in the ‘lme4’ package in R (v. 4.0.3). For mixed models, we tested the significance of fixed effects with Likelihood Ratio Tests; in the absence of random effects, we tested fixed effects using Anova in the ‘car’ package. Finally, we used Mantel tests to determine whether genetic relatedness (pairwise F_st_ values, Table S1) explained differences in any of the host traits at each of the three temperatures.

**Table 1:** Source information for genotypes of *Symbiodinium microadriaticum*.

Culture ID	Host species	Source location	Source lab
CCMP 2458	*Cassiopea andromeda*	Gulf of Aqaba, Indian Ocean	LaJeunesse
CCMP 2464	*Cassiopea xamachana*	Florida	LaJeunesse
FLCass	*Cassiopea xamachana*	Long Key, Florida	Coffroth
KB8	*Cassiopea* spp.	Kaneohe Bay, Hawaii	Coffroth
RT 362	*Cassiopea andromeda*	Gulf of Aqaba, Indian Ocean	LaJeunesse

## Supplementary Information

Supplementary Information.

## Data Availability

Data are archived and publicly available at the Biological and Chemical Oceanography Data Management Office (BCO-DMO): Physiology data (https://doi.org/10.26008/1912/bco-dmo.874597.1), in vitro growth data (https://doi.org/10.26008/1912/bco-dmo.874619.1), host fitness data (https://doi.org/10.26008/1912/bco-dmo.874609.1).
