# Supplementary material for: Symbiont genotype influences holobiont response to increased temperature
Source: Sci Rep. 2022 Nov 1;12:18394. doi: 10.1038/s41598-022-23244-3 (PMC9626619; doi:10.1038/s41598-022-23244-3)
Supplement: Supplementary file 1 — Supplementary Information. [file 41598_2022_23244_MOESM1_ESM.docx]

**Supplementary Material**

**Table S1.** Pairwise F_ST_ differences among algal genotypes

|  | CCMP2458 | CCMP2464 | FLCass | KB8 |
| --- | --- | --- | --- | --- |
| CCMP2458 (Gulf of Aqaba) |  |  |  |  |
| CCMP2464 (Florida) | 0.227 |  |  |  |
| FLCass (Florida) | 0.229 | 0.153 |  |  |
| KB8 (Hawaii) | 0.220 | 0.144 | 0.145 |  |
| RT362 (Gulf of Aqaba) | 0.148 | 0.216 | 0.238 | 0.211 |

| Trait | 26°C | 30°C | 32°C |
| --- | --- | --- | --- |
| Respiration | 0.212 | -0.333 | 0.176 |
| Gross Photosynthesis | -0.273 | -0.491 | 0.067 |
| Net Photosynthesis | -0.236 | -0.564 | 0.249 |
| Growth Rate (r) round 1 | -0.055 | 0.236 | 0.261 |
| Growth Rate (r) round 2 | 0.261 | 0.249 | 0.018 |
| Carrying Capacity (K) round 1 | -0.491 | 0.236 | -0.067 |
| Carrying Capacity (K) round 2 | **0.624*** | 0.127 | **0.709*** |
| Time to Infection | 0.103 | 0.065 | 0.103 |
| Time to Strobilation | -0.018 | -0.055 | -0.200 |
| Time to Ephyra Production | -0.018 | 0.249 | -0.200 |
| Number of Buds | 0.358 | -0.018 | -0.091 |
| Number of Ephyrae | 0.532 | -0.055 | -0.049 |

**Table S2**. Mantel r values of spearman matrix correlations comparing differences in trait values measured at 26, 30, and 32°C with genetic differences based on F_ST_ values in Table S1. Significant correlations (P<0.05) are marked in bold with *.

**Figure S1**
